# Science for the sustainable use of ecosystem services

**DOI:** 10.12688/f1000research.9470.1

**Published:** 2016-11-02

**Authors:** Elena M. Bennett, Rebecca Chaplin-Kramer

**Affiliations:** 1 Department of Natural Resource Sciences and McGill School of Environment, McGill University, Ste-Anne-de-Bellevue, Canada; 2 Natural Capital Project, Stanford University, Stanford, USA

**Keywords:** sustainable development, human wellbeing, human impact, conservation

## Abstract

Sustainability is a key challenge for humanity in the 21st century. Ecosystem services—the benefits that people derive from nature and natural capital—is a concept often used to help explain human reliance on nature and frame the decisions we make in terms of the ongoing value of nature to human wellbeing. Yet ecosystem service science has not always lived up to the promise of its potential. Despite advances in the scientific literature, ecosystem service science has not yet answered some of the most critical questions posed by decision-makers in the realm of sustainability. Here, we explore the history of ecosystem service science, discuss advances in conceptualization and measurement, and point toward further work needed to improve the use of ecosystem service in decisions about sustainable development.

## Introduction

As human societies continue to transform the biosphere, sustainability—the ability for humans to continue to exist and even thrive on the planet—has emerged as a key challenge. Ecosystem services (ESs)—the benefits that people derive from nature and natural capital—is a concept often used to help explain human reliance on nature ^1^ or to justify conservation actions ^2^. At the same time, the use of ESs by people (for example, food, fresh water for drinking, and recreation) is a necessary part of improving human wellbeing (HWB) ^3^, and some of those uses are consumptive, impacting future provision of the service or the continued provision of other services. This ultimately leads to a situation in which society must determine how best to use ESs to improve HWB now while working to ensure that ESs will be provided equitably now and for future generations. This article examines the state of the science of ESs and considers what more we still need to learn to help ensure that ESs are being used sustainably.

The concept of ESs has become hugely popular, in part because it frames the decisions we make affecting nature in terms of the value of nature to people (including values beyond monetization) and makes those values more visible ^4, 5^. The ES concept also has the potential to bring together issues of sustainability and environment with those of international development, which has been discussed for quite some time in the environment and development literatures but has proven difficult to achieve ^6, 7^. ESs are also quantifiable, and differences in their provision thus can be related to biophysical or ecological aspects of landscapes, to land use and management decisions, or to changes in values, individual preferences, and social demand, making the concept ideal for those who aim to improve conservation while taking development into consideration.

Yet, over the years, there have been many articles criticizing the ES concept or wondering why the concept and data about ESs have not led to more sweeping changes to decision-making ^2, 8– 10^. Indeed, despite considerable advancement in this field over the past few decades, ES science is still not answering some of the most critical questions posed by decision-makers in the realm of sustainability ( Table 1). In this article, we explore the history of ESs, discuss advances in conceptualization and measurement, and point toward advances needed to improve the use of ES in decisions about sustainable development.

**Table 1.** Future directions in ecosystem service science and their importance.

Future direction	Key question asked by decision-maker	Why it is important	How it could be done	Example citations
Future directions in the long term				
Focus on ecosystem service (ES) provision over longer time frames.	How will decisions made now play out in terms of ES and human wellbeing (HWB) now, in 10 years, and in 100 years?	Sustainability is about the long term; snapshot measures of ESs are of limited benefit for assessing sustainability.	Integration of models of climate and other drivers of long-term change and ES outcomes. Use existing historical data.	98, 99
Understand the role of path dependency and legacies in the provision of ESs.	How will decisions made now constrain future ES provision, even if different or better decisions are made later?	Current land use may create legacies that affect future ES provision because of path dependencies.	Study role of past land use in provision of services today.	56, 100
Plan for sustainable use of ESs in a changing—not static—world.	How will climate change or global economic change affect the ES outcomes of land use decisions?	Climate, diets, biogeochemical cycles, increasing prevalence of plastics—we know the world is changing.	Uncertainty analysis of different scenarios of change for more robust decision-making	29
Future directions of sustainable social-ecological systems				
Understand implications of ES provision for HWB.	How will livelihoods, human health, or HWB be impacted by a change in ES resulting from a decision (and which ESs are the critical ones to consider)?	The relationships between ESs and HWB are often as complex as (or more complex than) the relationships between ecosystems and ESs, and better understanding of these linkages is needed.	Better integration of ES science with other disciplines: development economics, public health, psychology, and sociology	65, 66
Understand the role of multiple forms of capital in the provision of ES and HWB.	When can various forms of human capital substitute for natural capital in the provision of ES (and when can it not)?	It is difficult to know how important natural capital is without comparing its importance in service provision with other capitals.	Build models that estimate the role of multiple forms of capital in the provision of ESs.	60
Understand the function of social ecological systems in the provision of ESs, their resilience, and distribution.	How do decisions to build up or compromise different sources of capital affect the resilience of ES provision and HWB in the long term?	ESs are provided by social- ecological systems, not ecosystems alone. Leaving out the social system will result in incomplete or incorrect answers to our pressing questions.	Expand system boundaries of ES studies to include social systems in addition to ecological ones.	26, 43
Understand the role of teleconnections and trade in the provision of ESs and HWB.	When can ESs be purchased from elsewhere (for example, through international trade) and when must they be locally produced?	Trade is an increasingly important part of ecosystem function in a globalized world.	Expand system boundaries of ES studies to include traded goods.	101, 102
Future directions of decision-relevant information				
Measure and monitor ES governance and outcomes.	In order to answer the above examples of decision-maker questions, the science needs to be co-developed with practitioners and decision- makers, to be measured in a way that matters to people and that resonates for decisions, and to be tracked over time so we can continue to learn together and adapt our future questions and research.	Information is limited, so we must proceed with what we do know and measure effectiveness to adapt.	Funding for monitoring of both ecological and social outcomes as an integral part of policy implementation	64
More co-designed research, working with stakeholders from the start		Science will be more relevant to decisions and more easily adopted if stakeholders are involved.	Create incentives in academia for engaging decision-makers and create opportunities in policy for approaching management as an experiment.	79, 103
Improve understanding of indicators and clearer use of them.		Decision-makers need simple metrics to track progress and trends, even where data are scarce.	Explain the choice of indicator, rationale, and alternatives.	76

## Ecosystem services and sustainability science: key steps forward

Grappling with the human impact on the environment is not new; in some ways, the conversation about people, nature, and conservation is centuries old (for example, 11, 12). Yet the conversation has changed over time. Mace ^13^ showed how the framing of the conversation has shifted from a conservation that prioritized wilderness (1960s and before) to one that focused on threats to habitats (1970s and 1980s) to the emergence of ESs in the 1990s as a way to reveal the importance of nature to HWB and finally (currently) to a nuanced perspective that recognizes the complex, dynamic relationships between people and nature.

Throughout this conceptual evolution, scientists have weighed in on the importance of ecosystem process and function to the “life-support system” of earth and the role of conservation in maintaining this life-support system. The first use of the term “ecosystem services”, by Ehrlich and Ehrlich ^14^, actually built off an even earlier reference to “environmental services” ^15^, as Ehrlich himself later attributed ^16^. The Study of Critical Environmental Problems ^15^ provided examples of the functions and processes performed by ecosystems that are essential to life as we know it: pest control, pollination, soil retention, soil formation, nutrient cycling, atmospheric and climate regulation, flood control, and fisheries. Missing from this list are some of the benefits that resonate most with people today: water provision and purification, timber production and non-timber forest products, recreation and eco-tourism opportunities, forage production, and crop production. Many of these latter services are the so-called final services that are directly relevant to people, the production of which the initially defined list of services plays a critical role in supporting.

Early ecological literature on ESs continued to focus on the supporting services (for example, 17), which echoed the familiar-to-ecologists ecosystem processes but which often stopped just short of articulating the direct benefit to people, whereas the economics literature at the time sought to value nature in terms of its utility to humans as a way of internalizing externalities ^18^. When society bears the cost (the externality) of an activity that degrades an ecosystem for private benefit, it is difficult to incentivize (internalize) more sustainable behavior without an adequate accounting for the full cost of that degradation. The main challenge economists were beginning to grapple with in valuing nature by around the turn of the century was that the whole was greater than they could express through summing the parts; available methods for price setting and valuation focused on small (marginal) changes, while it was well understood that the life-support system provided by nature was priceless ^19^. So the question for economists became the impact of intermediate change, especially when an intermediate amount of change can intersect an ecological threshold that pushes the system into a new state, resulting in fairly small ecological changes that can lead to significant impact on the long-term provision of ESs ^20– 22^.

The soaring popularity of ESs really emerged in the ecological literature by the late 1990s ^1^, at which point the first hint of cultural values for ESs (“support of diverse human cultures” and “providing of aesthetic beauty and intellectual stimulation that lift the human spirit”) ^1^ were added to a conceptualization of services that was still very much oriented toward ecosystem functions, at least in the ecological literature (much of the environmental economics literature had picked up on these issues earlier but typically without the ecological function orientation). As the list of recognized ESs grew longer and more directly connected to HWB ^3, 23^, the relevance of the concept to policy and decision at a variety of levels became evident. Fundamentally, the concept of ESs grew out of a time when nature was ignored by many facets of human enterprise and scientists and conservationists desired a way to highlight the value of nature to society.

### Frameworks for assessing ecosystem services

**Figure 1.**
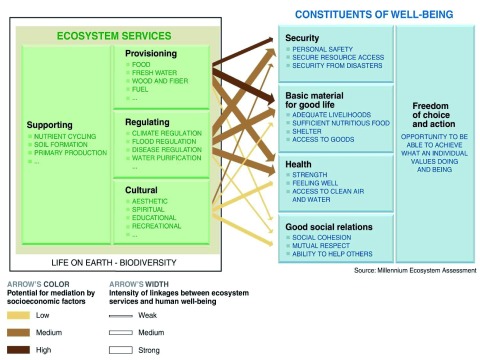
The Framework of the Millennium Ecosystem Assessment (2002). Reproduced from the Millenium Ecosystem Assessment (2002).

Around the turn of the century, scientists identified the need for an international assessment of ESs. The idea was to develop something akin to the Intergovernmental Panel on Climate Change but for ecosystems, taking advantage of recent advances in ecological and other sciences in the 1990s and putting this knowledge in the hands of international policy-makers ^24^. Ultimately, this became the Millennium Ecosystem Assessment (MA). The MA developed an ES framework that reflected the thinking of the time, showing a relationship between ESs (left side of Figure 1) and HWB (right side of Figure 1); arrows generally flowed from ecosystems and their services toward wellbeing, and the focus was on understanding the intensity of linkages and the potential for mediation of the relationship ^3, 23^.

**Figure 2.**
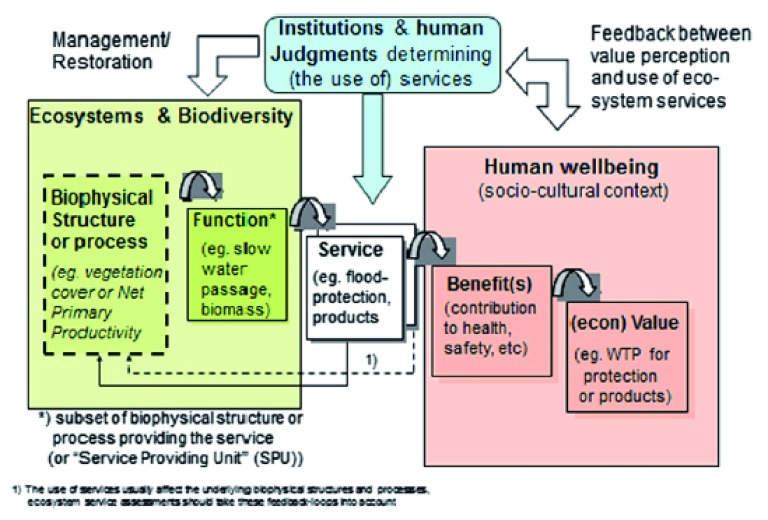
The Economics of Ecosystems and Biodiversity Framework. WTP, willingness to pay. Reproduced from Braat and de Groot ^78^ (2012), adapted from Haines-Young and Potschin ^25^ (2010).

In an update conceived by de Groot *et al*. ^5^ and based on the cascade model of Haines-Young and Potschin ^25^, the MA framework is broken down into more of its constituent parts ( Figure 2). Here, ecosystem structure and functions (that occupy much of the early thinking on ESs noted in the previous section) support the provision of a service, which is the actual benefit of nature to people. Breaking down the concept into these parts helps to gain clarity on *how* services are provided by nature, allowing the ecosystem to become multi-faceted and complex in our understanding. De Groot *et al*. ^5^ have added here the idea of feedbacks from HWB to both ESs and ecosystems, indicating that people can alter ecosystems and the services provided on the basis of their desires or differences between their perception of services provided and services required. Although we know that people are critical players in the delivery of ESs—plowing, fertilizing, seed planting, and harvesting are needed to produce food, even if there is already good natural capital (for example, high-quality soils) in place—the primary idea of even this new framework is that ecosystems alone ultimately provide services to people. That is, there is a general and primary flow from the left (ecosystems) to the right (people), and feedbacks are from the people to the ecosystem.

**Figure 3.**
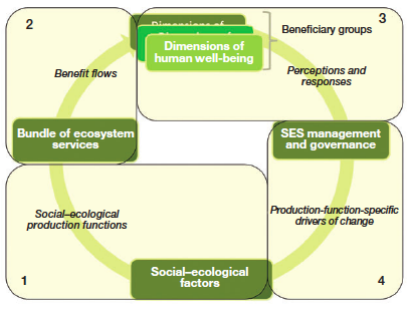
A social-ecological framework for ecosystem service and human wellbeing. SES, social-ecological systems. Reprinted with permission from Reyers *et al*. ^26^ (2013).

This rather linear conceptualization of ESs was given another significant update ^26^ ( Figure 3), including a diagram in which it is not “nature” but social-ecological systems (nature and people together), which generate bundles of services (multiple services together), which impact various dimensions of HWB. HWB, in turn, affects management and governance. In this framework, the social system plays a critical role not only in demanding and receiving ESs but also in determining their provision. This is significant because it emphasizes the role of society in the provision of services, moving the ES conversation to the more nuanced depiction of dynamic human-nature relationships ^13, 27, 28^. Also critical in this framework is the explicit consideration of ES bundles rather than single services. Other non-linear frameworks have been proposed. For example, Liu *et al*. ^29^ note that telecoupling (socioeconomic and environmental interactions between different places) is increasingly important in the Anthropocene and ought to be included in the way we understand and measure ESs.

### Advances in mapping and measuring multiple services

Beyond frameworks, the past decade has also brought considerable advances in measuring ESs and using these measurements as tools in decision-making. One of the most critical and basic advancements brought about by ES science is the focus on the ways that we use ecosystems (actively and passively) to ensure HWB ^28^ and the (direct and indirect) contribution of ecosystems to HWB ^30, 31^. A major challenge in this has been in approaching the science of ESs as holistically as the concept implies, especially in terms of interactions between multiple services, spatial dynamics, and change over time.

Although some early articles focused on mapping multiple ESs to understand associations among services ^32^ or among multiple services and biodiversity (for example, 33, 34), much of the ES literature has failed to live up to the promise of ESs to link nature and HWB, or to consider all aspects of sustainability, and a great many articles examine only one service, measure services at a single time snapshot, or measure ecosystem function with little or no connection to HWB ^35^. By contrast, decision-makers most often need information about multiple services and how they affect outcomes for people in order to make decisions that improve long-term sustainability ^5^. When the goal is to use ESs to improve sustainable development, considering multiple services over time is critical because any decision is likely to result in trade-offs, in which increases in some services lead to or are associated with declines in production of other services ^36^.

However, there are examples of ES studies that have focused on multiple services and sometimes on linking these to potential decisions or to HWB. For example, Bateman *et al*. ^37^ highlight the UK National Ecosystem Assessment, which combined spatially explicit ecological models with economic valuation to assess ESs and improve land use planning in the UK. Scientists have looked for areas that are hot spots of high provision of multiple services and understanding what creates these win-wins (for example, 32– 34). Qiu and Turner ^38^, for example, found that, although most relationships among ESs were synergies, hot spots (locations where at least six services were produced at high levels) were quite rare, occupying just 3.3% of the landscape. These authors also found that trade-offs were not consistent across space. That is, whereas most locations had a trade-off between crop production and water quality, some locations had both high water quality and high crop production, suggesting that this trade-off is not inevitable or that it might be able to be ameliorated. This study not only provided specific data about ES provision in a particular place for decision-makers in that region but also developed some generalizable knowledge, such as that managing ESs over large areas is likely to improve our ability to manage for sustainable provision of services ^38^.

A recent development in ES sciences is the movement from studies of single services or pairs of services to studies that focus on bundles of ESs, associations of several ESs that appear together repeatedly in space or time. Queiroz *et al*. ^39^ identified five distinct types of ES bundles in Sweden: “forests and towns”, “remote forest”, “mosaic cropland-livestock”, “mosaic cropland-horse”, and “urban”. They found that the production of an ES or ES bundle was based on a combination of the social-ecological potential (ecological and biophysical conditions and management practices) of a landscape to produce it and the human demand for that same service. In this as in many studies on ES bundles, hot spots of provisioning service production are often cold spots for regulating services. Thus, it is increasingly apparent that both ecological and social gradients are needed to adequately predict patterns of ES provision across landscapes ^40– 45^.

In addition to understanding how social-ecological landscapes provide ES bundles, progress has been made in refining the idea of trade-offs among pairs of services. Mouchet *et al*. ^46^ suggest that there are three types of trade-off assessments. Supply-supply assessments are about how supply of one service affects supply of another; supply-demand assessments are about whether the ecosystem can meet demand; and demand-demand assessments are about stakeholders’ competing interests. The method the authors propose takes a step toward embracing social-ecological systems and complexity in considering both supply and demand as well as their interactions. The importance of understanding the difference between supply, demand, and ecosystem capacity to provide services was also highlighted by Villamagna *et al*. ^47^, who pointed out that it is the comparison of supply with demand in the current time that determines satisfaction with the services provided and that it is the comparison of capacity with supply that determines the potential for sustainability.

Another advance in measuring and mapping ESs has been work to understand the role of landscape structure—the configuration of land use and land cover—in the provision of services. This is important for landscape management, especially over large areas, as suggested by Qiu and Turner, as the structure of the landscape is something that land use planners can influence with reasonable precision. Different spatial patterns of land use and cover may benefit different services, and trade-offs between services can be mitigated or exacerbated by fragmentation at different scales ^48, 49^. Landscape complexity, measured in terms of the proportion or diversity of natural habitat or its arrangement in the areas surrounding agriculture or both, enhances the pollination of crops ^50^ and pest control by natural predators ^51^ in a variety of cropping systems and regions ^52^. For these mobile organisms benefitting cropland, increasing interface between human and natural systems generally increases service provision, assuming an adequate ratio of natural to agricultural habitat. Other services may be reduced by this type of natural-agricultural mosaic; Chaplin-Kramer *et al*. ^53^ demonstrate that forest fragmentation reduces carbon sequestration by an average of 25% across the tropics. Whereas previous studies had estimated carbon sequestration on the basis of a benefits-transfer approach that assumes a direct relationship between forest area and carbon storage, the study by Chaplin-Kramer *et al*. shows that the configuration of the land use, and especially the amount of forest edge created by deforestation, makes a difference to the amount of service provided. Studies of carbon storage in temperate forests also find important effects of both spatial structure and human management ^54^. For water-related services, the aspect of landscape configuration that matters most is hydrologic connectivity to water courses ^55^, which presents yet another type of spatial habitat pattern to consider in managing for multi-functional landscapes.

Although many ES studies have been snapshots of service provision at a single moment in time, it is widely acknowledged that ESs and their relationships with each other, and with the drivers that affect their supply, are unlikely to remain constant over time ^46^. Recent studies have begun to examine how changes in landscapes over time have affected the provision of services. Renard *et al*. ^56^ found that some individual service relationships varied through time, shifting from trade-offs or no relationship at the start of the study (1971) to synergies by the end (2006). In terms of bundles, in 1971, the landscape was diverse, but most municipalities across the landscape were all providing similar bundles; by 2006, the landscape remained fairly diverse, but each municipality tended to specialize in the provision of one or a few services.

Finally, mapping and measuring ESs have been and will continue to be critical methods for understanding past trends and anticipating future trajectories and for evaluating the impact of policy interventions and whether goals are being met. However, many decisions require more information than can be gleaned from examining changes that have already occurred; policy-makers and other incentive setters wishing to promote the sustainable use of ESs have questions about where the most important places are to invest in conservation or restoration, what the impacts of allowing different types of development will be for different stakeholders, or how to most cost-effectively mitigate those impacts ( Table 1). In many cases, measuring ESs on the ground in specific places has led to empirical models that can inform decision-makers in those regions ^48, 57^. For example, work by Fisher *et al*. ^58^ focused on understanding the various benefits of reducing deforestation in Tanzania.

### Including people, governance, and resilience

These improvements in scientists’ ability to map, measure, and model individual ESs, pairs of ESs, and bundles of ESs have led to discussions about how ES science could be used to improve ecosystem management or to understand and assess progress toward policy targets such as the Sustainable Development Goals. However, actual use of ES science in decision-making has sometimes fallen short of expectations ^2, 59, 60^. Carpenter *et al*. ^9^ suggest that part of the problem may be a discipline-bound focus of some of the science as well as failure to consider feedbacks over a range of biophysical and social systems. Reyers *et al*. ^26^ propose that, in the rush to gather data, science has found itself with a plethora of measures that fall short of their intended purpose. That is, we have lots of measurements, but these use conflicting definitions or poor indicators, which makes use of ESs to assess targets like the Sustainable Development Goals difficult at best. Newer studies are advancing rapidly in developing multiple ES-based methods for assessing the effect of policy interventions on sustainability. For example, Hamann *et al*. ^61^ developed and tested an approach to mapping social-ecological systems on the basis of characteristic bundles of ES use. The idea was that these bundles ultimately could be used as a tool to identify different social-ecological system types and target governance interventions more appropriately.

One outcome of the focus on social-ecological systems has been increased interest in HWB and in co-design of research projects with stakeholders, research that actively involves decision-makers to improve uptake and use of information in decision-making ^62^. Cundill and Fabricius ^63^ suggest that although there is a wealth of frameworks for understanding complex systems, relatively less attention has been given to co-design and other approaches that might facilitate learning as part of the monitoring process. Such co-design is important because it can help ensure that science is both interesting to the scientific community and useful to decision-makers ^64^. This helps mainstream ESs as a part of decision-making. Similarly, researchers are now pointing out the need to include more nuanced, quantitative analyses of ESs and HWB ^65^ and even developing indices to understand when and how wellbeing depends on provision of services ^66^. Ultimately, if decision-makers are concerned with the wellbeing of constituents or stakeholders, research that goes only as far as the biophysical components of services may be less useful than work that quantifies HWB outcomes or seeks to understand relationships between services and wellbeing.

Including social-ecological systems has also paved the way to merge concepts of resilience with ESs, a significant step forward in moving ES science to a place where it can improve ecosystem management for sustainability. Extensive anthropogenic changes, mostly to provide certain ESs such as food, have increased the likelihood of large, non-linear, and possibly irreversible changes in ecosystems ^67, 68^. Recognizing that enhancing ES resilience is crucial for meeting current and future societal needs, Biggs *et al*. ^69^ use the literature to suggest seven principles for enhancing the resilience of ESs in social-ecological systems: maintain diversity and redundancy, manage connectivity, manage slow variables and feedbacks, foster an understanding of social-ecological systems as complex adaptive systems, encourage learning and experimentation, broaden participation, and promote polycentric governance systems. Developing this definitive set of resilience-enhancing principles for ESs and a synthetic understanding of when and where they apply is important for those in the governance arena seeking to ensure a sustainable provision of services for the long term while avoiding large, non-linear changes in service provision, which can have catastrophic effects on HWB.

## Continuing issues

Several countries, including the UK, China, and South Africa, have undertaken national ecosystem assessments ^37, 70– 72^, which are an important step toward measuring and monitoring change in ESs on a large scale. Findings from such efforts are being translated to diverse contexts in many other countries through international collaborations such as the Intergovernmental Panel on Biodiversity and Ecosystem Services (IPBES) or the Group on Earth Observations–Biodiversity Observation Network (GEOBON). Experimental accounting methods—for example, promoted by the Wealth Accounting and Valuation of Ecosystem Services (WAVES) program—at institutions like the World Bank aim to transform national accounts to reflect ES values in countries’ financial ledgers. However, most of these examples are focused on assessing current baselines or past trends, and although this is an important first step to integrating ES into decisions, it does not provide action-ready and easily digestible information about the consequences of any particular land, ocean, or natural resource use decision. Although regional and national ecological and economic assessments are helpful, they do not typically help mainstream the type of decision support that is now needed across scales.

Furthermore, although models exist for including ES in a cost-benefit analysis or return-on-investment calculation, too often models are not accessible enough at the time they are needed, or data are not available to complete them, with the result that ESs are often left out of environmental impact reports or development or zoning plans. Part of this is due to a failure on the part of the scientific community to standardize data and approaches in forms that could be used by agency staff or consulting groups they contract to make it standard practice. In a promising new development, the US has recently mandated all federal agencies to consider how ESs could be included in their planning ^73^, and with some trial and error this may help to better articulate what form such standard approaches should take.

Additionally, despite advances, many gaps remain in our understanding of the ecology of ESs, some of which were already recognized more than a decade ago ^74^, and the field still faces multiple challenges: a fairly discipline-bound science ^9^, limited involvement of stakeholders in the development of the questions that guide many scientific studies ^75^, and confusion about the multiple potential indicators available for measuring ESs ^76^. For these and perhaps other reasons, ES science has not been fully adopted by decision-makers and has not reached the full potential of socio-ecological integration it is intended to promote ^77^.

One of the original agendas of ES researchers was to bring together natural science and economics, conservation, and development ^78^. Although conceptually ESs seemed poised to make this happen, ES researchers have not yet been entirely successful. Many efforts remain bound by disciplinary boundaries. This may be, in part, because the types of questions that are interesting differ among disciplines in ways that present significant barriers to funding and publishing.

Involving stakeholders in question development, and sometimes in the scientific process of answering those questions, may also hold important keys for improving the use of ES science in decision-making. In fact, it is so important that some have found that stakeholder involvement in ES research is a better predictor of policy change than the nature of the findings themselves ^79^. This may be because one benefit of co-designed science is the trust that is built between scientists and decision-makers ^80^ or because it encourages scientists to work at the scale at which decisions are being made ^81^.

There also remains considerable confusion in the use of terms, especially in the use of indicators. The difference between ecosystem process, function, and service remains muddled, and some scientists use these terms interchangeably whereas others make clear distinctions. Liss *et al*. ^76^ surveyed the literature on pollination as an ES and found that, in 121 studies that self-identified as being about both ESs and pollination, 62 unique metrics were used to assess the amount of pollination. The authors showed how these inconsistent measurements complicate attempts at comparison or synthesis and can even lead to very different management decisions.

Ultimately, many are finding that, for a variety of reasons, ecology as it stands is limited in its predictive capacity to identify the sustainable use of any particular ES and to describe the trade-offs between uses of ESs ^82– 84^. Moving from one ecosystem to another or, better yet, developing general principles ^4^ has been difficult ^85^. Some general principles emerging from this work are beginning to be included in broadly accessible, globally applicable tools for decision support and scenario analysis, such as Integrated Valuation of Ecosystem Services and Tradeoffs (InVEST) ^86^, Artificial Intelligence for Ecosystem Services (ARIES) ^87^, and others ^88, 89^ (see 90 for a review of these tools). The goal of these tools is to improve access of decision-makers to the latest ES knowledge in an easily digestible format that allows better integration of ESs into decisions affecting the sustainable use and provision of ESs in the future. However, these tools are still challenged by data constraints for model parameterization and calibration to make them fully accessible and accurate and are lacking representation of key components (for example, threshold effects) or services (especially cultural). That is, it is still difficult to know, for any particular land use or management decision, the outcomes in both the short and long term for a full set of relevant ESs. And although we do have the ability to determine this with scads of research in a particular location, this often takes longer than the time frame in which the decision must be made. Thus, the ES concept has proven useful in moving forward thinking, but the science has not been sufficiently predictive to support full application of the concept in decision-making for sustainability.

Despite such limitations, from a value-of-information perspective, the knowledge of ESs we are currently capable of representing is adequate for informing many types of decisions, including spatial targeting of best management practices ^91, 92^ or restoration opportunities ^93^, understanding equity implications of development impacts ^94^, and identifying where green infrastructure has the potential to support or enhance grey infrastructure ^95^. Although many unanswered questions remain in defining and planning for truly sustainable development, we can learn from implementing policies based on the science we do have, tracking outcomes, and adaptively managing for improvement ^96, 97^.

## Future directions

For ESs to contribute to enhancing the sustainability of the human enterprise, ES science must move beyond conceptualizing and mapping and into addressing the difficult, complex questions that have dogged sustainability science for decades. Based on the review of the literature here, we propose a handful of critical future directions for ES science ( Table 1).

The future directions we suggest include many that effectively expand the boundaries of ES research in several directions. We suggest that ES science consider longer time frames and include ongoing global change, non-linearities, and uncertainty in this analysis. ES science should also expand its disciplinary boundaries to include information about multiple forms of capital and the role of social systems in demand for, and provision of, services and include stakeholders in design and even implementation of some research plans. Expanding physical boundaries to include the role of trade in moving some types of services around the planet will also be a critical next step in understanding planetary ES provision and its implications for sustainability.

ES science holds great promise to help society assess progress toward meeting planetary sustainability and development goals such as the Sustainable Development Goals. To fully achieve that potential and to answer the most important questions decision-makers are asking, the science must continue to develop beyond disciplinary boundaries to consider the long-term provision of ESs by social-ecological systems.

## Abbreviations

ES, ecosystem service; HWB, human wellbeing; MA, Millennium Ecosystem Assessment.
